# Genome-wide identification and salt stress response analysis of the WD40 protein family in *Populus yunnanensis*

**DOI:** 10.1186/s12864-026-12560-x

**Published:** 2026-01-23

**Authors:** Yi Wu, Yude Kang, Lincui Shi, Aizhong Liu, Ping Li

**Affiliations:** 1 Key Laboratory of Forest Resources Conservation and Utilization in the Southwest Mountains of China (Ministry of Education), College of Forestry, Southwest Forestry University, Kunming, China; 2 Key Laboratory for Conservation and Utilization of in Forest Resource of Yunnan, Southwest Forestry University, Kunming, China; 3 College of Biological Science and Food Engineering, Southwest Forestry University Kunming, Kunming, China

**Keywords:** WD40 proteins, *Populus yunnanensis*, Salt stress, Phylogenetic analysis, Protein interaction network

## Abstract

**Background:**

The WD40 proteins constitute a large regulatory family involved in a wide range of biological processes and stress responses. Advances in sequencing technologies have facilitated the identification of numerous WD40 proteins with diverse functions in many plant species. However, research on tree species remains limited. *Populus yunnanensis* is an economically and ecologically important tree species that faces salt stress in certain habitats. Therefore, studying WD40 proteins in *P. yunnanensis* is of particular significance for understanding its salt tolerance mechanisms.

**Results:**

A variable number of WD40 proteins were identified across six poplar species, generally correlating with their genome sizes. In *P. yunnanensis*, 258 PyWD40s were identified, exhibiting considerable variation in amino acid number, and other physicochemical properties, suggesting potential functional diversity. The chromosomal distribution of these genes was uneven, indicative of gene family formation and replication events. High collinearity among members and low Ka/Ks values suggested strong sequence conservation and purifying selection. Notable similarities were also observed between the WD40 proteins of *P. yunnanensis* and *A. thaliana*. Structural analysis revealed that representative PyWD40s from the eight subfamilies could be modeled into three-dimensional structures rich in β-sheets. Variations in conserved domains and exon numbers contributed to sequence and functional divergence. Expression profiling under salt stress indicated that most PyWD40s were upregulated under both short-term and prolonged stress conditions, a finding validated by qRT-PCR for 14 selected genes. Protein–protein interaction network analysis demonstrated that PyWD40s interact with proteins involved in process such as signal transduction, and their co-expression under salt stress suggests a coordinated role in facilitating the stress response.

**Conclusions:**

In summary, this study provides comprehensive insights into the roles of PyWD40 proteins in the salt stress response of *P. yunnanensis*. These findings contribute significantly to the understanding of stress tolerance mechanisms in this species and offer a valuable resource for future functional studies.

**Supplementary Information:**

The online version contains supplementary material available at 10.1186/s12864-026-12560-x.

## Introduction

The WD40 domain represents an ancient and extensive family of regulatory proteins in eukaryotes. This family is characterized by conserved core repeat units, each typically terminating with a Trp-Asp (WD) dipeptide sequence [1]. As one of the most abundant protein–protein interaction domains in eukaryotic genomes, the WD40 domain plays a vital role in mediating interactions between protein and DNA [2, 3]. Structurally, the domain assumes a β-propeller architecture, generally composed of seven blades, with each blade comprising four antiparallel β-strands [4, 5]. This domain folds into a β-propeller structure within proteins, offering a platform for the interaction and assembly of multiple protein partners, often serving as a scaffold with signalosome [3]. The β-propeller structure provides three distinct interaction surfaces (top, bottom, and circumference), enabling WD40 proteins to engage in reversible, multi-partner binding. A well-characterized example is the Gβ subunit of heterotrimeric G proteins, which forms a tightly associated dimer (Gβγ) with the Gγ subunit. Due to the central role of G proteins in transmembrane signaling, WD40 domains are frequently, though often misleadingly, labeled as “transducing-like” [6]. The functional repertoire of WD40 proteins encompasses a broad spectrum of critical eukaryotic processes. Certain WD-repeat proteins, functionally analogous to Gβγ, are integral to signal transduction cascades, serving as subunits of phosphatase [7], anchors for protein kinase C, or adaptors for receptor complexes [8]. The conserved three-dimensional structure common to WD40 family members underlies similar binding interfaces and functional capacities, implying evolutionary and mechanistic links to G-protein signaling pathways [9].

WD40 proteins constitute a functionally diverse group with roles spanning various organisms. In yeast, for instance, several WD-repeat proteins perform specific functions. The *Saccharomyces cerevisiae* protein Pfs2p forms multi-protein complexes essential for mRNA cleavage and polyadenylation [10]. Additionally, the WD-repeat proteins PWP2 and PEB1 are important for cell separation and the import of thiolase into peroxisomes [11, 12]. Futherthmore, WD40 repeat motifs are genetically related to those of the TPR (tetratricopeptide repeat) family, highlighting an evolutionary connection between these widespread repeat-protein families [12]. In plants, WD40 proteins exhibit similarly diverse functions. In *Arabidopsis thaliana*, the WD40 domain cyclophilin, CYP71 acts as a histone remodeling factor involved in chromatin-based gene silencing, contributing to gene repression and organogenesis regulation [13]. *Arabidopsis* XIW1, an XPO1-interacting WD40 protein, interacts with XPO1 and ABI5, functioning as a nuclear transport receptor that positively regulates the ABA (abscisic acid) response [14]. In rice, a WD40 protein OsKRN2 regulates rice grain number by controlling secondary panicle branching and exhibits a conserved interaction with DUF1644 in both maize and rice [15]. The rice *WD40* gene *OsTTG1* participates in biosynthetic pathways through interactions with numerous proteins, particularly transcription factors [16]. Similarly, in mango, the WD40 protein MiTTG1 interacts with MiMYB0, MiTT8, and MibHLH1 to form a ternary MYB-bHLH-WD40 complex, enhancing tolerance to mannitol, salt, and drought stress when expressed in transgenic *Arabidopsis* [17]. In peppers, interactions between WD40 proteins and CaAN1 or CaDYT1 implicate them in anthocyanin biosynthesis and male sterility [18]. In maize, the WD40 protein SHREK1 contributes to ribosome biogenesis and kernel development via interactions with other ribosomal proteins [19]. Moreover, WD40-repeat proteins can act as substrate recognition subunits for DDB1-CUL4 ubiquitin E3 ligase complexes, thereby influencing diverse biological processes [20]. Collectively, these examples underscore that protein–protein interactions are a fundamental mechanism through which WD40 proteins exert their wide-ranging biological functions across different organisms.

Extensive research on WD40 proteins across various plant species has primarily focused on their identification, quantification, and phylogenetic classification. In cereal crops, for example, 743 WD40 proteins were identified in wheat (*Triticum aestivum* L.) and classified into 5 clusters comprising 11 subfamilies [21]. Similarly, 164 HvWD40 proteins in barley (*Hordeum vulgare* L. var. nudum Hook. f, also known as Qingke) were grouped into 11 clusters and 14 subfamilies [22], 225 *WD40* genes in foxtail millet (*Setaria italica* L.) were categorized into 5 subfamilies (I—V) [23], and approximately 200 potential *OsWD40* genes were identified in rice [24]. Among solanaceous plants, 168 WD40 proteins in potato (*Solanum tuberosum* L.) were divided into 5 clusters (Cluster I-V) and 10 classes [25], while 207 *WD40* genes in tomato (*Solanum lycopersicum* L.) were classified into 5 clusters and 12 subfamilies [26]. The number of WD40 proteins in different pepper (*Capsicum annuum*) species ranged from 237 to 257 [18]. In horticultural plants, 315 WD40 members were identified in mango (*Mangifera indica* L.) and further divided into 11 subgroups [17], and 191 WD—repeat (WDR) proteins in cucumber were classified into 21 subgroups [27]. For tree species, a more limited study identified 42 JrWD40s in walnut (*Juglans regia* L.), classifying them into 9 clusters [28].

WD40 repeat proteins are crucial components of eukaryotic genomes, participating in diverse developmental processes and environmental interactions in plants. A seminal study in maize delineated a nearly complete pan-genome WD40 repertoire, revealing substantial functional and genomic diversity and suggesting that gene duplications and Helitron transposon-mediated translocations have played key roles in the amplification of this gene family [29]. Collectively, these studies underscore the widespread distribution and functional versatility of WD40 proteins across the plant kingdom. However, the majority of this research has been conducted on herbaceous model and crop species, leaving a significant gap in our understanding of WD40 proteins in woody plants, particularly forest trees. Given the profound ecological and economic importance of forest trees, a deeper exploration of their WD40 protein family is essential. *Populus yunnanensis*, a tree species native to the low latitudes and high-altitude regions of southwest China, represents one such economically and ecologically important species [30–32]. In this context, our study aims to conduct the identification, bioinformatics analysis, and exploration of stress resistance of WD40 proteins in *P. yunnanensis*, which will contribute to a more comprehensive understanding of the functions and regulatory mechanisms of WD40 proteins in woody plants.

## Results

### WD40 proteins showed significant differences among poplar species

WD40 proteins were identified in *Populus* species via a homology-based search for the conserved WD40 domain. Subsequent validation using the NCBI CD-search and SMART databases confirmed the presence of WD40 proteins in six *Populus* species: 258 in *P. yunnanensis*, 250 in *Populus trichocarpa*, 427 in *Populus tomentosa*, 380 in *Populus euphratica*, 357 in *Populus alba*, and 270 in *Populus deltoides* (Table S1). This analysis revealed considerable variation in WD40 protein copy number across the examined *Populus* species.

We conducted a detailed analysis of the physicochemical properties of the 258 identified WD40 proteins in *P. yunnanensis*, designated PyWD40-1 to PyWD40-258 based on their gene IDs (which correspond to their chromosomal order; Table 1). These proteins exhibited substantial variation in amino acid length (103–3600 residues) and molecular weight (11,640.07 to 400,117.3 Da). The theoretical isoelectric points (pI) ranged from 4.32 to 9.68, classifying 80 as basic (pI > 7) and 178 as acidic (pI < 7). Using an instability index threshold of 40, we found that the number of unstable proteins (156; instability index > 40) was greater than that of stable ones (102; instability index < 40), with stability indices themselves panning a wide range (21.1 (PyWD40-164) to 64.67 (PyWD40-226)). The aliphatic index also varied considerably (49.96–103) further indicating diversity in protein stability. Most PyWD40 proteins were hydrophilic, with grand average of hydropathicity (GRAVY) values between −0.906 and −0.02. Only three proteins (PyWD40-24, PyWD40-85, PyWD40-235) were predicted to be hydrophobic, with positive GRAVY values of 0.128, 0.032, and 0.028, respectively. Subcellular localization predictions suggested diverse cellular roles, with 141 proteins localized to the nucleus, 55 to the chloroplast, 47 to the cytoplasm, 6 to the plasma membrane, 3 each to mitochondria and the cytoskeleton, 2 to the vacuole, and 1 to the peroxisome.

**Table 1:** Physicochemical characteristics and subcellular location of *P. yunnanensis* WD40 proteins

Protein ID		Gene ID	Number of amino acids	Domain	Molecular weight (Da)	Isoelectric point (pI)	Instability index	Aliphatic index	GRAVY	Subcellular localization
PyWD40-1	Poyun00230		416	73–397	45,403.81	8.54	42.27	86.9	−0.108	Cytoplasm
PyWD40-2	Poyun00272		765	189–470	85,126.37	8.71	58.95	49.46	−0.872	Nucleus
PyWD40-3	Poyun00457		349	35–304	38,776.82	8.6	41.97	64.81	−0.444	Nucleus
PyWD40-4	Poyun00467		773	488–773	84,010.78	6.42	42.11	68.93	−0.519	Nucleus
PyWD40-5	Poyun00710		505	381–462,289–462	56,211.67	8.51	52.82	76	−0.417	Nucleus
PyWD40-6	Poyun00746		458	15–248	51,806.2	5.35	46.36	81.97	−0.152	Cytoplasm
PyWD40-7	Poyun00814		746	265–587	84,091.66	6.22	50.29	72.45	−0.476	Nucleus
PyWD40-8	Poyun00900		1069	287–1069	118,842.49	5.8	45.33	73.08	−0.54	Nucleus
PyWD40-9	Poyun00943		1522	560–715	166,176.34	5.83	42.2	87.81	−0.041	Nucleus
PyWD40-10	Poyun01093		530	207–506	58,358.97	9.24	44.36	78.94	−0.269	Chloroplast
PyWD40-11	Poyun01755		892	297–653,93–452,32–123	99,738.35	6.33	40.21	86.45	−0.206	Chloroplast
PyWD40-12	Poyun01768		350	13–300	38,003.5	5.59	43.52	73	−0.364	Nucleus
PyWD40-13	Poyun01819		916	17–297	103,483.67	4.93	34.79	84.72	−0.343	Chloroplast
PyWD40-14	Poyun02059		1129	416–724,578–1020,939–1109	124,493.63	7.82	39.42	83.99	−0.23	Chloroplast
PyWD40-15	Poyun02184		948	66–226	106,280.45	5.09	45.52	89.51	−0.271	Nucleus
PyWD40-16	Poyun02265		350	13–345	38,687.19	4.82	29.95	73.8	−0.295	Nucleus
PyWD40-17	Poyun02714		1138	12–335	122,779.25	5.09	49.24	79.78	−0.277	Chloroplast
PyWD40-18	Poyun02740		211	12–190	23,155.4	9.06	31.78	68.39	−0.317	Chloroplast
PyWD40-19	Poyun03087		342	19–305	37,888.48	8.51	42.9	68.42	−0.48	Cytoplasm
PyWD40-20	Poyun03185		547	28–362	59,738.91	4.95	32.5	90.71	−0.24	Nucleus
PyWD40-21	Poyun03193		300	14–299	33,110.18	6.45	36.35	80.5	−0.286	Cytoplasm
PyWD40-22	Poyun03587		503	65–386	55,923.21	8.41	48.88	72.13	−0.454	Nucleus
PyWD40-23	Poyun03591		342	4–308	37,762.6	5.86	49.76	74.97	−0.245	Cytoplasm
PyWD40-24	Poyun03954		412	2–324	43,587.65	5.67	43.79	95.15	0.128*	Cytoplasm
PyWD40-25	Poyun04097		903	15–903	101,202.92	5.75	58.54	77.56	−0.391	Nucleus
PyWD40-26	Poyun04131		969	482–803	106,421.56	5.44	46.87	71.08	−0.616	Nucleus
PyWD40-27	Poyun04214		429	128–429	47,705.06	7.51	51.01	81.96	−0.307	Nucleus
PyWD40-28	Poyun04247		371	100–365	41,681.46	5.72	51.78	81.91	−0.142	Cytoplasm
PyWD40-29	Poyun04659		325	72–241	35,606.68	7.03	42.37	75.6	−0.206	Nucleus
PyWD40-30	Poyun04764		313	13–295	35,193.67	6.15	26.35	77.57	−0.397	Cytoplasm
PyWD40-31	Poyun04773		445	242–425	50,036.41	5.03	29.96	65.71	−0.533	Nucleus
PyWD40-32	Poyun04963		675	361–669	75,183.69	6.26	53.79	76.87	−0.45	Nucleus
PyWD40-33	Poyun05016		1098	346–673,848–1059	120,991.47	6.63	37.11	84.03	−0.255	Chloroplast
PyWD40-34	Poyun05093		308	17–294	34,910.67	5.8	28.65	80.65	−0.227	Cytoplasm
PyWD40-35	Poyun05117		902	614–902	98,926.18	6.33	50.79	68.58	−0.649	Nucleus
PyWD40-36	Poyun05204		479	111–432	54,685.38	8.84	50.07	74.68	−0.375	Nucleus
PyWD40-37	Poyun06131		1545	1121–1373	173,898.67	6.47	51.32	86.6	−0.235	Nucleus
PyWD40-38	Poyun06144		501	145–403	55,285.76	5.73	46.34	72.61	−0.549	Nucleus
PyWD40-39	Poyun06199		321	12–319	34,271.74	5.92	29.21	80.56	−0.128	Cytoplasm
PyWD40-40	Poyun06355		548	291–510	58,689.48	7.66	41.54	72.03	−0.341	Nucleus
PyWD40-41	Poyun06433		959	403–699,102–520	106,432.08	6.88	42.85	87.92	−0.311	Chloroplast
PyWD40-42	Poyun06658		1280	52–427,409–732	139,122	6.02	43.43	78.76	−0.255	Nucleus
PyWD40-43	Poyun06783		940	397–722	104,481.19	8.58	53.48	71.46	−0.598	Nucleus
PyWD40-44	Poyun06973		1131	399–692,763–1051	124,694.11	6.57	40.95	80.05	−0.326	Nucleus
PyWD40-45	Poyun07194		574	264–562	63,513.73	5.37	45.7	80.61	−0.446	Nucleus
PyWD40-46	Poyun07580		448	254–428	50,876.48	4.95	34.47	67.86	−0.534	Nucleus
PyWD40-47	Poyun07710		725	384–723	82,549.13	6.17	42.44	73.93	−0.715	Nucleus
PyWD40-48	Poyun08098		1043	11–361	113,937.55	6.02	36.38	78.8	−0.317	Nucleus
PyWD40-49	Poyun08183		355	49–355	39,497.48	5.24	39.12	72.76	−0.32	Nucleus
PyWD40-50	Poyun08219		417	168–395	46,467.59	4.58	31.85	81.37	−0.44	Cytoplasm
PyWD40-51	Poyun08357		515	201–504	57,888.99	9.05	43.91	73.2	−0.538	Nucleus
PyWD40-52	Poyun08421		377	57–369	41,133.1	6.52	31.89	78.33	−0.222	Nucleus
PyWD40-53	Poyun08457		761	12–294	84,142.11	5.66	34.92	98.21	−0.167	Cytoplasm
PyWD40-54	Poyun08477		457	106–426	50,649.26	9.11	36.83	78.62	−0.384	Chloroplast
PyWD40-55	Poyun09470		318	61–310	35,698.31	5.46	37.51	81.23	−0.142	Cytoplasm
PyWD40-56	Poyun09610		657	370–656	73,559.9	7.27	45.6	73.65	−0.498	Nucleus
PyWD40-57	Poyun10044		543	72–539	62,547.92	6.27	49.27	83.11	−0.388	Vacuole
PyWD40-58	Poyun10283		780	503–780	85,723.15	6.67	50.9	67.44	−0.558	Nucleus
PyWD40-59	Poyun10360		449	250–429	50,707.26	4.95	43.69	73.41	−0.449	Cytoplasm
PyWD40-60	Poyun10513		424	124–404	48,400.03	4.72	44.43	78.87	−0.547	Nucleus
PyWD40-61	Poyun10528		346	96–296	39,054.71	4.76	51.71	83.41	−0.427	Cytoskeleton
PyWD40-62	Poyun10560		347	13–342	38,688.17	4.91	30.56	73.31	−0.343	Nucleus
PyWD40-63	Poyun10673		442	149–338	48,349.33	6.26	47.27	75.23	−0.311	Nucleus
PyWD40-64	Poyun10691		384	29–193	42,005.85	5.39	41.47	76.15	−0.32	Nucleus
PyWD40-65	Poyun10857		1402	129–380	156,523.67	5.98	42.68	91.19	−0.118	Nucleus
PyWD40-66	Poyun11002		744	271–578	82,425.99	5.81	46.35	75	−0.343	Nucleus
PyWD40-67	Poyun11008		2542	2220–2482,2444–2539	279,553.24	6.22	43.28	88.24	−0.077	Nucleus
PyWD40-68	Poyun11256		2983	2254–2511	325,592.51	5.48	50.2	88.03	−0.145	Plas membrane
PyWD40-69	Poyun11277		438	83–431	47,628.47	9.01	26.9	86.42	−0.265	Chloroplast
PyWD40-70	Poyun11451		485	120–440	52,949.85	8.84	39.15	73.15	−0.412	Nucleus
PyWD40-71	Poyun11518		276	50–156	31,083.48	5.09	43.7	90.4	−0.097	Cytoplasm
PyWD40-72	Poyun11600		819	15–330,533–704	90,811.9	6.48	43.66	83.08	−0.267	Nucleus
PyWD40-73	Poyun11671		3268	664–1084	366,516.41	5.95	43.54	96.56	−0.109	Plas membrane
PyWD40-74	Poyun11750		103	44–92	11,640.07	5.4	48.85	95.53	−0.296	Chloroplast
PyWD40-75	Poyun11838		472	275–452	53,671.26	5.18	28.15	77.88	−0.38	Chloroplast
PyWD40-76	Poyun12238		580	261–560	64,522.32	6.04	47.35	93.59	−0.263	Nucleus
PyWD40-77	Poyun12281		314	16–311	34,890.6	8.61	29.23	81.91	−0.224	Mitochondrion
PyWD40-78	Poyun12329		3595	3311–3591	399,637.05	5.7	47.28	93.45	−0.133	Plas membrane
PyWD40-79	Poyun12363		139	2–112	15,045.11	8.72	41.24	77.99	−0.284	Cytoplasm
PyWD40-80	Poyun12596		444	176–444	48,790.03	7.51	42.22	93.06	−0.022	Cytoplasm
PyWD40-81	Poyun12699		348	36–348	38,748.27	4.95	41.48	80.17	−0.287	Nucleus
PyWD40-82	Poyun13009		1503	1235–1503	167,765.03	5.82	48.83	88.89	−0.241	Chloroplast
PyWD40-83	Poyun13258		3600	3317–3596	400,117.3	5.71	46.78	93.69	−0.137	Plas membrane
PyWD40-84	Poyun13292		432	29–243	46,577.01	5.32	26.67	93.1	0.032*	Chloroplast
PyWD40-85	Poyun13342		581	262–560	64,849.68	5.98	45.03	94.11	−0.273	Nucleus
PyWD40-86	Poyun13718		304	118–295	33,058.55	5.51	32.57	87.3	−0.03	Chloroplast
PyWD40-87	Poyun13808		758	41–331,634–708	83,770.91	6.27	56.46	74.26	−0.488	Nucleus
PyWD40-88	Poyun13907		317	12–313	34,825.27	7.2	30.1	81.1	−0.217	Peroxisome
PyWD40-89	Poyun13953		573	220–531	64,846.38	5.13	27.26	70.24	−0.706	Nucleus
PyWD40-90	Poyun14057		541	179–499	60,123.13	9.29	49.89	81.76	−0.264	Vacuole
PyWD40-91	Poyun14231		332	21–313	36,943.81	6.19	31.91	81.3	−0.218	Cytoplasm
PyWD40-92	Poyun14619		477	161–400	52,683.52	5.14	47.78	72.6	−0.504	Nucleus
PyWD40-93	Poyun14762		503	157–475	56,050.3	5.92	51.52	70.36	−0.773	Chloroplast
PyWD40-94	Poyun14796		655	208–522	74,020.37	6.26	49.78	68.66	−0.527	Nucleus
PyWD40-95	Poyun14896		761	34–366	83,390.4	6.8	34.99	83.89	−0.285	Chloroplast
PyWD40-96	Poyun14939		989	350–494	107,334.5	5.55	61.36	75.55	−0.307	Chloroplast
PyWD40-97	Poyun15045		382	202–279,33–279	42,744.99	8.63	37.48	86.44	−0.115	Nucleus
PyWD40-98	Poyun15101		1127	340–659,835–1086	124,597.59	7.14	38.42	83.43	−0.269	Chloroplast
PyWD40-99	Poyun15326		615	12–229	67,335.92	5.79	36.88	91.32	−0.242	Nucleus
PyWD40-100	Poyun15420		480	162–457	53,023.11	9.11	40.13	71.08	−0.464	Nucleus
PyWD40-101	Poyun15512		474	115–455	53,337.84	7.6	44.66	77.3	−0.462	Nucleus
PyWD40-102	Poyun15770		433	99–418	47,527.02	9.06	37.82	94.97	−0.211	Chloroplast
PyWD40-103	Poyun15907		630	275–574	71,113.35	6.87	38.32	67.51	−0.559	Nucleus
PyWD40-104	Poyun15922		524	226–513	56,979.22	6.16	27.12	82.46	−0.281	Nucleus
PyWD40-105	Poyun16274		326	2–314	35,629.14	6.37	33.32	78.04	−0.311	Chloroplast
PyWD40-106	Poyun16276		301	8–285	32,641.47	5.86	24.34	80.37	−0.255	Chloroplast
PyWD40-107	Poyun16464		833	57–444,377–830	91,928.41	6.04	40.3	85.91	−0.14	Nucleus
PyWD40-108	Poyun16687		1713	222–631	192,306.18	5.97	48.51	66.05	−0.736	Nucleus
PyWD40-109	Poyun16797		1674	1257–1672	184,584.2	6.48	49.72	91.62	−0.124	Nucleus
PyWD40-110	Poyun17261		485	189–461	52,710.15	9.25	43.88	73.11	−0.42	Nucleus
PyWD40-111	Poyun17550		385	116–307	42,495.6	6.18	36.78	85.82	−0.142	Chloroplast
PyWD40-112	Poyun17551		537	122–344	60,054.4	9.53	26.77	76.67	−0.528	Cytoplasm
PyWD40-113	Poyun17794		505	207–501	56,954.95	5.37	56.27	103.27	−0.114	Cytoplasm
PyWD40-114	Poyun17805		748	9–245	81,705.82	6.89	33.57	84.43	−0.26	Chloroplast
PyWD40-115	Poyun17872		727	386–725	82,829.4	6.31	45.04	71.72	−0.734	Nucleus
PyWD40-116	Poyun18136		459	102–364,327–456	50,493.65	5.6	49.32	80.46	−0.375	Chloroplast
PyWD40-117	Poyun18265		463	117–444	51,128.16	8.94	45.66	84.04	−0.193	Chloroplast
PyWD40-118	Poyun18316		911	17–297	102,948.15	4.92	36.55	85.28	−0.323	Chloroplast
PyWD40-119	Poyun18356		350	13–299	37,908.29	5.4	43.03	72.69	−0.347	Nucleus
PyWD40-120	Poyun18365		889	294–650,32–410	99,177.55	5.88	39.31	85.11	−0.187	Chloroplast
PyWD40-121	Poyun18967		492	143–468	53,805.39	9.25	46.98	74.35	−0.404	Chloroplast
PyWD40-122	Poyun19105		1513	550–715,16–120	165,387.55	5.95	42.91	86.73	−0.072	Nucleus
PyWD40-123	Poyun19149		1110	328–1110	122,761.65	5.4	41.95	74.48	−0.482	Nucleus
PyWD40-124	Poyun19218		746	266–588	83,749.24	6.36	44.26	75.32	−0.448	Nucleus
PyWD40-125	Poyun19276		458	15–248	51,732.08	5.32	43.69	82.6	−0.143	Cytoplasm
PyWD40-126	Poyun19533		785	500–785	85,404.15	6.44	43.88	67.75	−0.544	Nucleus
PyWD40-127	Poyun19544		501	187–456	55,162.45	9.22	43.39	66.79	−0.423	Nucleus
PyWD40-128	Poyun19713		757	184–465	83,616.69	8.05	57.93	53.3	−0.783	Nucleus
PyWD40-129	Poyun19717		750	177–458	83,359.45	8.71	55.13	51.08	−0.839	Nucleus
PyWD40-130	Poyun19757		419	75–399	45,795.36	8.81	39.88	82.1	−0.111	Cytoplasm
PyWD40-131	Poyun19991		445	242–425	50,292.6	4.92	31.12	67.24	−0.58	Nucleus
PyWD40-132	Poyun20013		1359	192–507	147,883.87	5.42	54.03	85.94	−0.276	Chloroplast
PyWD40-133	Poyun20069		512	202–512	57,738.99	6.81	41.9	82.46	−0.336	Nucleus
PyWD40-134	Poyun20399		942	455–776	103,625.18	5.82	47.39	68.34	−0.656	Nucleus
PyWD40-135	Poyun20435		905	15–905	101,593.7	5.45	57.2	79.88	−0.339	Nucleus
PyWD40-136	Poyun20792		337	31–160	36,525.61	9.46	35.81	88.19	−0.166	Chloroplast
PyWD40-137	Poyun20920		375	2–375	40,592.27	8.08	35.15	83.92	−0.145	Cytoplasm
PyWD40-138	Poyun20943		318	49–309	35,746.31	5.46	36.73	79.37	−0.171	Chloroplast
PyWD40-139	Poyun21117		990	29–293	108,835.26	8.24	48.53	80.7	−0.324	Plas membrane
PyWD40-140	Poyun21138		435	68–383	48,858.77	4.74	46.72	65.72	−0.511	Nucleus
PyWD40-141	Poyun21465		569	103–565	64,203.45	6.01	53.97	78.12	−0.426	Chloroplast
PyWD40-142	Poyun21574		808	9–245	88,255.82	7.29	36.39	82.38	−0.31	Chloroplast
PyWD40-143	Poyun21578		529	221–515	59,811.19	5.5	45.61	96.35	−0.198	Cytoplasm
PyWD40-144	Poyun21817		557	122–357	62,226.33	9.68	27.38	83.38	−0.442	Cytoplasm
PyWD40-145	Poyun21818		387	54–311	42,655.69	5.36	38.68	85.09	−0.163	Nucleus
PyWD40-146	Poyun22099		485	191–461	52,799.32	9.37	42.93	73.71	−0.422	Nucleus
PyWD40-147	Poyun22608		1729	185–644	193,966.38	6.28	49.12	65.76	−0.72	Nucleus
PyWD40-148	Poyun22966		301	19–285	32,929.62	5.36	23.56	77.08	−0.359	Chloroplast
PyWD40-149	Poyun22967		326	8–314	35,738.25	6.55	35.17	77.45	−0.352	Chloroplast
PyWD40-150	Poyun23268		525	226–514	57,182.64	6.22	25.66	84.34	−0.266	Nucleus
PyWD40-151	Poyun23280		581	282–581	65,676.74	6.33	39.52	65.3	−0.693	Nucleus
PyWD40-152	Poyun23525		389	112–326	43,897.32	7.2	53.95	74.68	−0.492	Nucleus
PyWD40-153	Poyun23794		465	157–442	51,245.28	9.26	37.28	77.14	−0.378	Nucleus
PyWD40-154	Poyun23796		1333	1039–1118	150,669.62	6.9	48.4	90.05	−0.275	Nucleus
PyWD40-155	Poyun24087		1124	337–656,440–947,832–1083	124,082.98	7.04	37.72	83.83	−0.27	Chloroplast
PyWD40-156	Poyun24149		382	202–279,55–279	42,479.49	7.63	34.34	87.96	−0.083	Cytoplasm
PyWD40-157	Poyun24241		989	372–408,353–494	107,146.35	5.6	57.31	75.65	−0.307	Chloroplast
PyWD40-158	Poyun24286		796	34–368	87,341.97	7.56	36.5	83.61	−0.266	Mitochondrion
PyWD40-159	Poyun24500		455	154–429	50,469.51	8.24	50.57	74.22	−0.547	Chloroplast
PyWD40-160	Poyun24786		868	26–611	95,289.49	5.9	40.97	89.75	−0.118	Nucleus
PyWD40-161	Poyun24805		1136	350–659,812–1103	125,050.98	6.63	43.27	80.72	−0.299	Nucleus
PyWD40-162	Poyun25188		389	53–386	41,603.74	4.38	35.42	81.7	−0.239	Cytoplasm
PyWD40-163	Poyun25281		153	2–153	17,271.8	7.77	21.1	70.72	−0.487	Cytoplasm
PyWD40-164	Poyun25717		651	179–498	71,677.18	5.35	45.82	68.33	−0.653	Nucleus
PyWD40-165	Poyun25719		519	212–509	58,475.46	5.55	44.4	88.98	−0.258	Nucleus
PyWD40-166	Poyun25852		424	110–403	48,406.99	4.7	42.52	77	−0.562	Nucleus
PyWD40-167	Poyun26057		672	110–403	75,830.7	7.08	47.88	75.57	−0.477	Nucleus
PyWD40-168	Poyun26169		443	249–423	50,078.88	5.18	37.26	71.51	−0.46	Nucleus
PyWD40-169	Poyun26226		378	17–232	42,369.74	7.56	37.3	75.77	−0.27	Nucleus
PyWD40-170	Poyun26235		346	96–296	39,038.75	4.75	51.42	84.83	−0.403	Cytoskeleton
PyWD40-171	Poyun26281		488	131–469	54,966.79	6.99	44.48	73.48	−0.411	Nucleus
PyWD40-172	Poyun26287		534	53–338	59,462.51	9.41	45.87	88.46	−0.268	Nucleus
PyWD40-173	Poyun26326		441	146–335	48,102.05	5.8	49.69	81.79	−0.247	Nucleus
PyWD40-174	Poyun26343		378	29–188	41,470.27	5.22	48.55	72.96	−0.327	Nucleus
PyWD40-175	Poyun26432		1611	233–614,577–661	180,839.93	6.83	46.66	70.97	−0.652	Nucleus
PyWD40-176	Poyun26578		1930	483–540,421–532	213,308.6	5.61	46.63	88.89	−0.248	Nucleus
PyWD40-177	Poyun26683		1051	251–1051	116,922.5	6.82	51.07	74.02	−0.483	Nucleus
PyWD40-178	Poyun26856		547	248–529	59,924.91	7.8	31.21	80.02	−0.246	Chloroplast
PyWD40-179	Poyun26914		2995	2249–2526	327,291.3	5.57	48.88	89.32	−0.166	Plas membrane
PyWD40-180	Poyun26947		435	79–427	47,344.09	9.02	27.94	86.55	−0.284	Chloroplast
PyWD40-181	Poyun27183		484	119–439	52,860.6	8.69	42.63	72.5	−0.413	Nucleus
PyWD40-182	Poyun27279		450	9–320	49,600.4	6.43	48.61	90.33	−0.188	Nucleus
PyWD40-183	Poyun27387		452	38–433	49,680.96	8.99	36.19	77.68	−0.38	Nucleus
PyWD40-184	Poyun27403		818	15–330,532–703	90,257.45	6.95	43.95	83.67	−0.261	Nucleus
PyWD40-185	Poyun27631		547	22–363	59,996.94	5.15	34.91	84.11	−0.34	Nucleus
PyWD40-186	Poyun27807		455	154–429	50,492.61	8.52	47.21	73.36	−0.552	Chloroplast
PyWD40-187	Poyun28071		770	4–505,505–543	85,458.75	5.94	36.03	89.87	−0.111	Nucleus
PyWD40-188	Poyun28315		833	533–829	92,260.86	5.8	36.25	88.85	−0.179	Cytoplasm
PyWD40-189	Poyun28547		390	54–387	41,803.65	4.32	36.29	78	−0.324	Cytoplasm
PyWD40-190	Poyun28629		345	47–342	38,381.51	7.18	39.13	68.35	−0.466	Nucleus
PyWD40-191	Poyun28659		315	47–312	34,989.66	6.59	35.5	70.54	−0.443	Nucleus
PyWD40-192	Poyun28974		452	59–360	51,680.73	9.48	45.05	68.83	−0.594	Nucleus
PyWD40-193	Poyun29223		658	208–521	74,084.18	5.96	44.99	69.38	−0.509	Nucleus
PyWD40-194	Poyun29246		451	3–357	49,430.21	7.23	42.22	85.9	−0.166	Nucleus
PyWD40-195	Poyun29250		503	157–475	56,166.7	6.29	56.17	71.33	−0.733	Nucleus
PyWD40-196	Poyun29381		481	184–404	52,755.52	5.01	52.2	72.83	−0.474	Nucleus
PyWD40-197	Poyun29891		575	222–533	64,907.51	5.33	25.36	68.99	−0.722	Nucleus
PyWD40-198	Poyun30065		467	100–426	51,639.4	9.39	49.98	74.09	−0.434	Mitochondrion
PyWD40-199	Poyun30228		489	208–474	53,324.49	4.52	42.47	81.96	−0.384	Nucleus
PyWD40-200	Poyun30331		317	32–313	34,789.17	6.58	33.88	74.95	−0.254	Cytoplasm
PyWD40-201	Poyun30406		365	38–304	39,642.48	4.89	49.23	78.3	−0.132	Cytoskeleton
PyWD40-202	Poyun30476		394	58–385	44,065.12	5.97	46.04	67.69	−0.498	Cytoplasm
PyWD40-203	Poyun30533		452	159–443	48,892.7	7.21	28.7	72.39	−0.297	Nucleus
PyWD40-204	Poyun30780		428	159–403	48,839.3	9.17	47	76.99	−0.592	Nucleus
PyWD40-205	Poyun30822		577	258–568	64,320.91	8.88	60.45	80.69	−0.569	Nucleus
PyWD40-206	Poyun30949		737	23–340	81,213.92	7.28	47.1	92.86	−0.099	Chloroplast
PyWD40-207	Poyun31013		509	217–508	56,133.31	5.8	36.86	87.41	−0.316	Nucleus
PyWD40-208	Poyun31190		406	61–400	44,962.81	8.04	36.37	90.99	−0.288	Nucleus
PyWD40-209	Poyun31200		1218	9–317	136,606.29	6.58	33.22	91.73	−0.201	Chloroplast
PyWD40-210	Poyun31213		301	8–285	32,719.52	5.67	22.42	80.33	−0.275	Cytoplasm
PyWD40-211	Poyun31425		1208	849–1145	134,170.98	5.93	45.07	89.92	−0.169	Chloroplast
PyWD40-212	Poyun31509		328	8–323	35,903.63	6.99	25.74	89.18	−0.124	Chloroplast
PyWD40-213	Poyun31513		609	66–607	66,137.93	6.26	30.18	88.64	−0.153	Chloroplast
PyWD40-214	Poyun31648		489	208–474	53,219.35	4.54	41.72	81.57	−0.38	Chloroplast
PyWD40-215	Poyun31814		723	270–579	80,860.56	5.58	48.87	67.37	−0.507	Nucleus
PyWD40-216	Poyun32038		452	64–362	51,657.71	9.39	44.2	71.39	−0.579	Nucleus
PyWD40-217	Poyun32609		340	19–307	37,510.25	5.81	48.52	77.12	−0.234	Nucleus
PyWD40-218	Poyun32678		494	137–450	54,064.44	6.67	33.87	83.1	−0.163	Nucleus
PyWD40-219	Poyun32813		439	115–432	48,181.49	5.5	37.57	89.48	−0.19	Cytoplasm
PyWD40-220	Poyun33524		722	473–690	78,759.82	7.07	29.33	81.11	−0.05	Nucleus
PyWD40-221	Poyun33545		188	3–156	21,013.81	5.25	27.6	76.17	−0.219	Nucleus
PyWD40-222	Poyun33617		675	361–669	75,234.67	6.32	51.87	78.18	−0.457	Nucleus
PyWD40-223	Poyun33651		1092	306–642,783–1016	121,126.13	5.27	41.64	85.93	−0.232	Chloroplast
PyWD40-224	Poyun33652		1128	421–688,836–1089	124,607.23	6.72	39.53	85.25	−0.207	Chloroplast
PyWD40-225	Poyun33766		987	699–987	110,019.41	6.56	64.67	60.2	−0.906	Nucleus
PyWD40-226	Poyun34148		503	150–408	55,378.92	5.94	48.33	71.73	−0.573	Nucleus
PyWD40-227	Poyun34648		1125	348–659,814–1045	123,920.31	6.75	39.21	79.8	−0.321	Chloroplast
PyWD40-228	Poyun35174		459	163–434	51,058.32	8.73	50.22	76.97	−0.506	Nucleus
PyWD40-229	Poyun35523		557	307–519	59,525.33	6.98	42.69	70.68	−0.337	Nucleus
PyWD40-230	Poyun35706		449	154–424	50,188.48	8.26	46.22	74.1	−0.48	Nucleus
PyWD40-231	Poyun35870		316	7–288	35,359.73	6.03	37.15	76.52	−0.315	Cytoplasm
PyWD40-232	Poyun35940		207	20–171	22,643.11	5.1	42.26	75.94	−0.229	Nucleus
PyWD40-233	Poyun35948		184	20–165	20,201.32	4.7	50.12	75.82	−0.246	Nucleus
PyWD40-234	Poyun36448		875	283–452	96,898.73	6.65	47.96	94.46	0.028*	Chloroplast
PyWD40-235	Poyun36485		728	273–584	81,324.87	5.35	47.93	72.01	−0.466	Nucleus
PyWD40-236	Poyun36778		609	66–607	65,990.84	6.19	27.09	90.08	−0.128	Cytoplasm
PyWD40-237	Poyun36783		328	8–323	36,045.88	7.6	27.24	87.68	−0.138	Cytoplasm
PyWD40-238	Poyun36884		1207	848–1144	133,938.46	5.97	43.52	89.66	−0.179	Nucleus
PyWD40-239	Poyun36943		968	27–300	106,491.96	6.09	38.33	81.49	−0.344	Nucleus
PyWD40-240	Poyun37040		1220	9–317	136,797.56	6.62	33.09	91.11	−0.202	Chloroplast
PyWD40-241	Poyun37054		403	58–397	44,455.91	7.02	35.1	90.94	−0.336	Nucleus
PyWD40-242	Poyun37368		582	263–573	64,813.46	9.24	60.64	81.36	−0.535	Nucleus
PyWD40-243	Poyun37635		450	157–441	48,899.92	7.22	28.92	73.78	−0.295	Nucleus
PyWD40-244	Poyun37688		390	64–385	43,448.3	5.74	45.76	70.15	−0.492	Cytoplasm
PyWD40-245	Poyun37840		387	9–382	41,943.21	5.96	36.1	80.39	−0.305	Cytoplasm
PyWD40-246	Poyun37843		950	64–224	107,037.41	5.1	42.61	89.71	−0.285	Cytoplasm
PyWD40-247	Poyun38349		1137	12–335	122,832.02	5.13	53.25	78.21	−0.299	Cytoplasm
PyWD40-248	Poyun38373		471	97–469	52,121.4	8.55	30.86	80.49	−0.332	Cytoplasm
PyWD40-249	Poyun38688		342	19–305	38,127.67	8.04	41.1	68.42	−0.495	Cytoplasm
PyWD40-250	Poyun38885		458	107–428	50,658.76	9.27	44.32	72.93	−0.503	Nucleus
PyWD40-251	Poyun38911		761	12–294	84,073.21	5.48	35.39	98.09	−0.15	Cytoplasm
PyWD40-252	Poyun38943		377	57–369	40,899.9	6.69	29.46	81.17	−0.173	Nucleus
PyWD40-253	Poyun39003		330	12–302	36,918.57	9.02	29.46	88.58	−0.02	Cytoplasm
PyWD40-254	Poyun39136		418	168–395	46,887.99	4.6	32.83	83.23	−0.468	Cytoplasm
PyWD40-255	Poyun39176		496	28–406	54,969.74	5.53	46.41	76.85	−0.39	Chloroplast
PyWD40-256	Poyun39226		1042	11–361	113,935.58	6.28	36.42	79	−0.33	Nucleus
PyWD40-257	Poyun39333		543	155–472	60,565.24	8.84	38.89	84.88	−0.262	Cytoplasm

The proteins PyWD40-8, PyWD40-25, PyWD40-58, PyWD40-124, PyWD40-136, PyWD40-142, PyWD40-168, PyWD40-178, PyWD40-203, and PyWD40-245 are reserved for the PLN00181 domain; the proteins PyWD40-69 and PyWD40-180 are reserved for the Beach domain; the protein PyWD40-74 is reserved for the Neurobeachin domain; the protein PyWD40-133 is reserved for the Ge1_WD40 domain. * Hydrophobic protein (GRAVY > 0). Proteins with a negative GRAVY value are hydrophilic and unmarked

### Phylogenetic analysis and sub-family classification of WD40 in *P. yunnanensis*

**Fig. 1:**
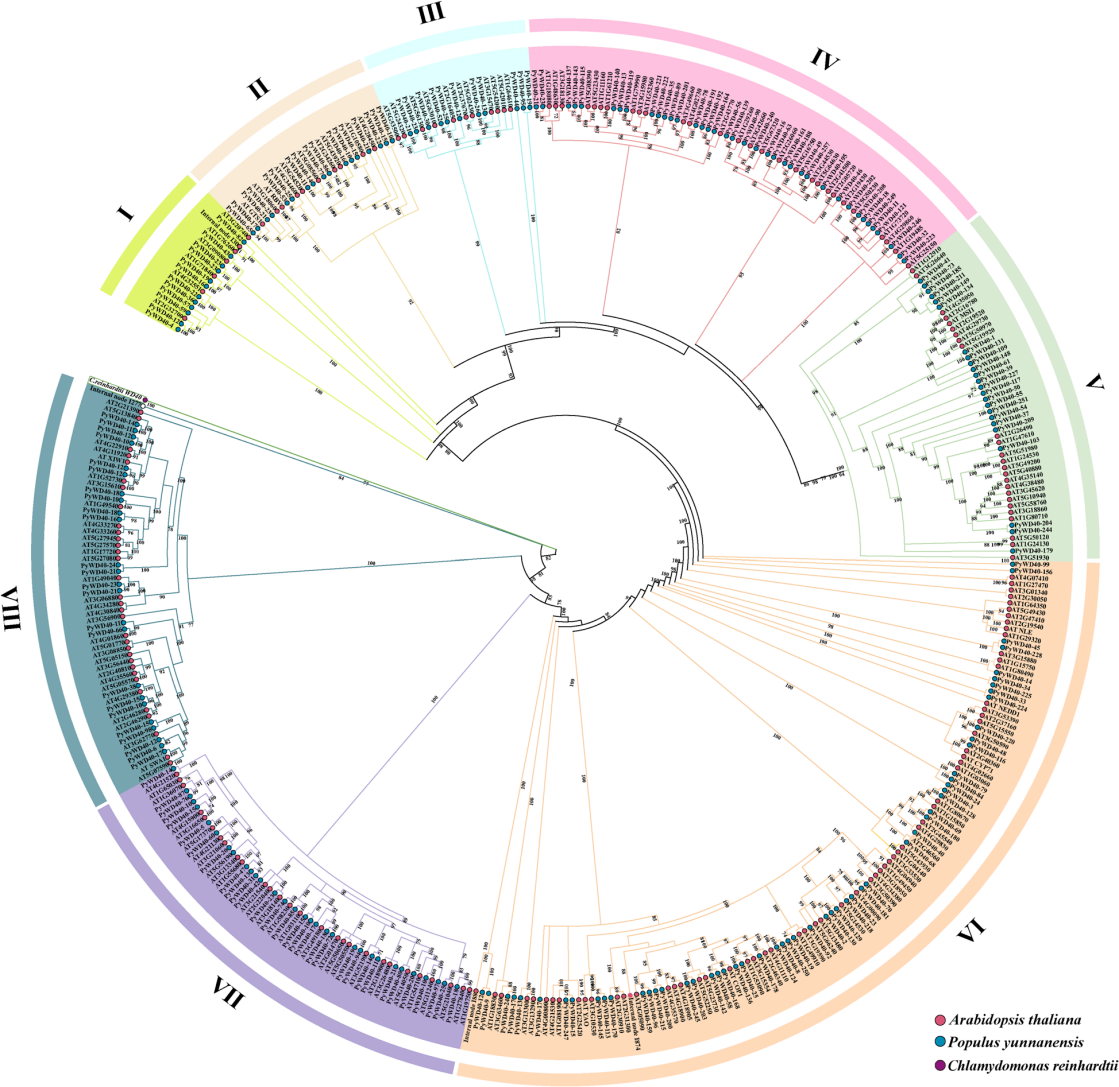
Phylogenetic analysis of the WD40 proteins from *P. yunnanensis* and *A. thaliana.* Protein sequences of WD40 from *P. yunnanensis* and *A. thaliana* were aligned using MAFFT and FastTree. The tree is rooted using *Chlamydomonas reinhardtii* (Cre06.g302750_4532, indicated by purple dots) as the outgroup. Protein origins are marked as follows: red dots, *A. thaliana*; blue dots, *P. yunnanensis*; purple dots, *C. reinhardtii*. Branch support values are based on 1000 bootstrap replicates

To elucidate the evolutionary relationships of WD40 proteins in *P. yunnanensis*, a phylogenetic tree was constructed using sequences from 258 *P. yunnanensis* and 230 *A. thaliana* WD40 proteins (Fig. 1). The proteins from both species clustered into eight major evolutionary clades. Following the established subfamily classification for *A. thaliana*, the *P. yunnanensis* WD40 proteins were correspondingly classified into eight subfamilies (I- VIII), containing14, 19, 13, 39, 49, 57, 34, and 33 members, respectively (Table S2).

### Uneven distribution and collinearity analysis of *WD40* on chromosomes in *P. yunnanensis*

**Fig. 2:**
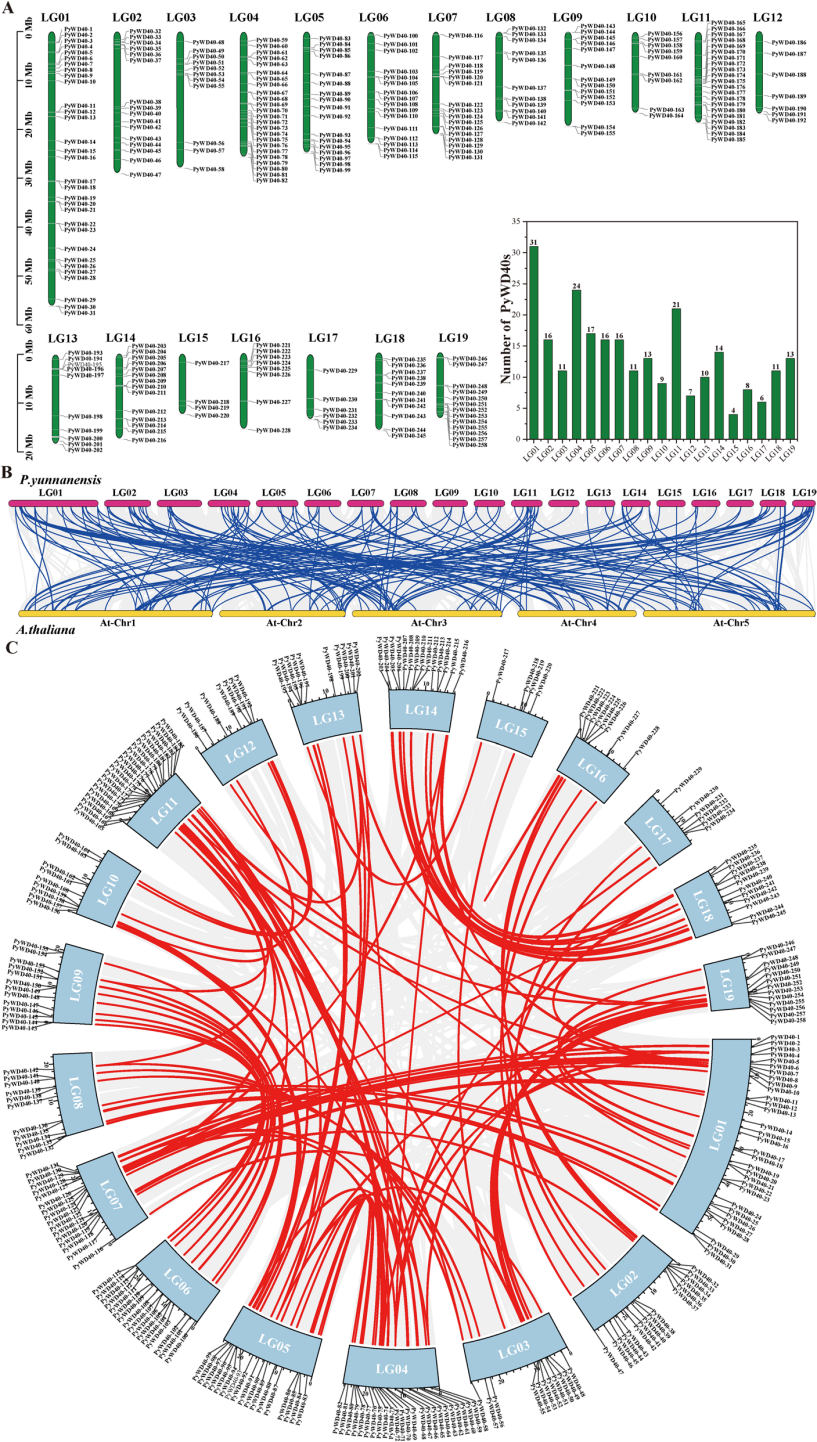
Chromosomal location and collinearity analysis of *WD40* genes from *P. yunnanensis*. **A** Chromosomal location of *WD40* genes in *P. yunnanensis*. The chromosome-level scaffolds are designated as “LG” (Linkage Group) according to the reference genome assembly. The green bar plot on the right displays the number of *WD40* genes located on each chromosome (LG). **B** Collinearity analysis of *WD40* genes from *P. yunnanensis* and *A. thaliana*. The blue lines indicate the collinearity relationship between *P. yunnanensis* and *A. thaliana WD40*s. The gray lines represent the collinearity relationships of all the genes in the genome of *P. yunnanensis* and *A. thaliana*. Violet boxes depict chromosomes of *P. yunnanensis*, yellow boxes depict chromosomes of *A. thaliana*. Chromosome numbers are indicated beside the boxes. **C** Intra-genomic collinearity analysis of *WD40* genes in *P. yunnanensis*. The red lines indicate the collinearity relationship among *P. yunnanensis WD40*s. The gray lines represent the collinearity relationships of all the genes in the *P. yunnanensis* genome

The 258 *PyWD40* genes were unevenly distributed across all 19 chromosomes of *P. yunnanensis* (designated LG01–LG19, where ‘LG’ denotes Linkage Group; Fig. 2A). Chromosome LG01 harbored the highest number (31 genes), followed by LG04 (24 genes) and LG11 (21 genes). LG05 contained 17 gens, while LG02, LG06, and LG07 each contained 16. The remaining chromosomes (LG03, LG08, LG09, LG10, LG12, LG13, LG14, LG16, LG17, LG18, LG19) contained 11, 11, 13, 9, 7, 10, 14, 8, 6, 11, and 13 genes, respectively. Chromosome LG15 contained the fewest, with only four *PyWD40* genes.

Collinearity analysis revealed 174 homologous pairs between *P. yunnanensis* and *A. thaliana WD40* genes (Fig. 2B, Table S3). Furthermore, intra-genomic analysis demonstrated strong sequence homology and conservation among *P. yunnanensis* WD40s identifying 114 segmental duplication pairs and one tandem duplication pair (*PyWD40-221* and *PyWD40-222*) (Fig. 2C, Table S4). The Ka/Ks ratios of all *WD40* gene pairs were all less than 1 (ranging from 0.0184 to 0.449), indicating that this gene family has undergone purifying selection and exhibits high sequence conservation (Table S5).

### Structural analysis of the three-dimensional models of WD40 proteins in *P. yunnanensis*

**Fig. 3:**
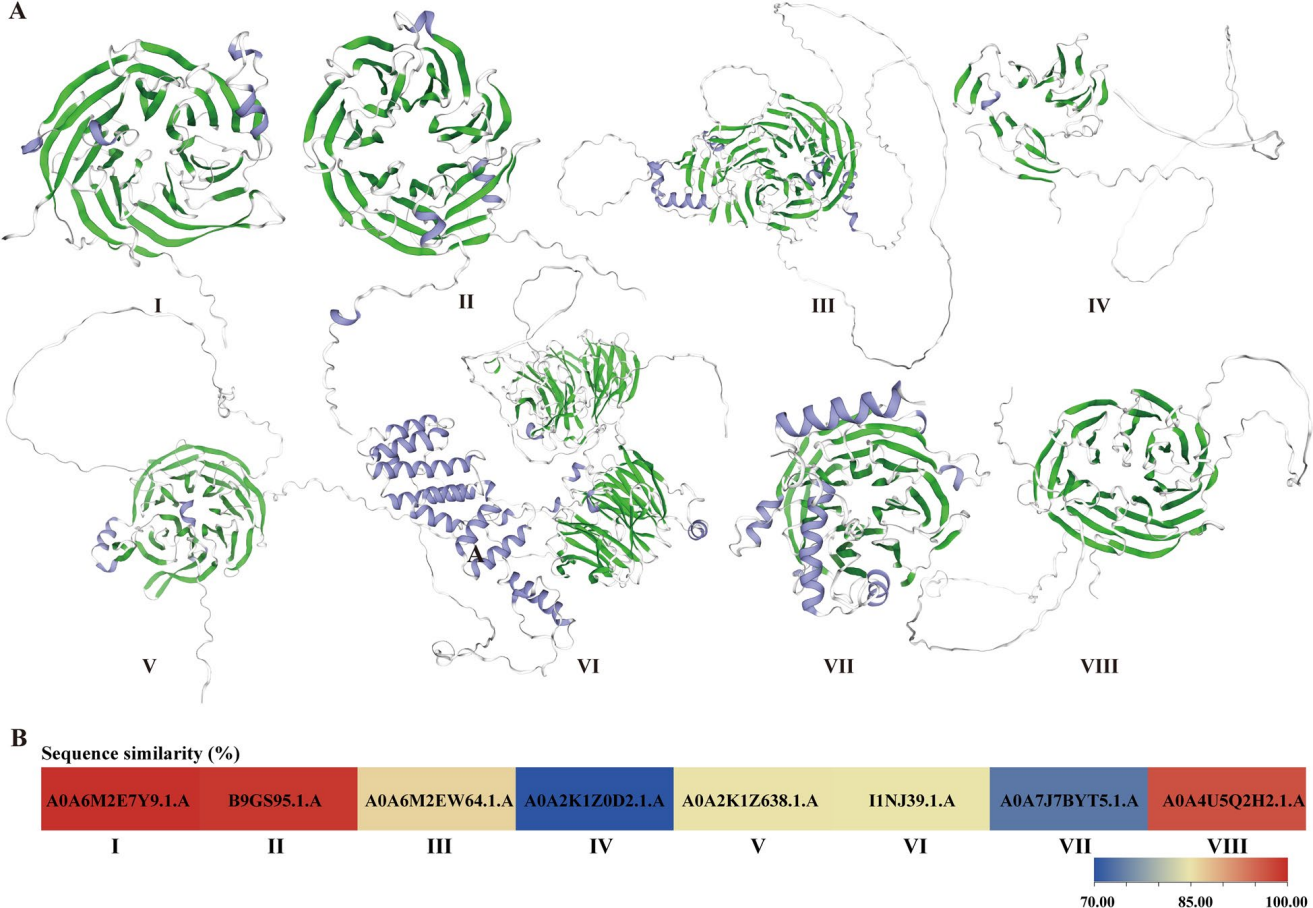
Protein 3D protein structure models and sequence similarity analysis of *P. yunnanensis* WD40s. **A** Predicted 3D structure models of the representative proteins from eight *P. yunnanensis* WD40 subfamilies. The models for PyWD40-28 (subfamily I), PyWD40-65 (subfamily II), PyWD40-216 (subfamily III), PyWD40-137 (subfamily IV), PyWD40-55 (subfamily V), PyWD40-99 (subfamily VI), PyWD40-85 (subfamily VII), and PyWD40-64 (subfamily VIII) were constructed based on the templates A0A6M2E7Y9.1.A, B9GS95.1.A, A0A6M2EW64.1.A, A0A2K1Z0D2.1.A, A0A2K1Z638.1.A, I1NJ39.1.A, A0A7J7BYT5.1.A, and A0A4U5Q2H2.1. **B** Heatmap depicting the sequence similarity between the *P. yunnanensis* WD40 proteins and their respective modeling templates. The color scale and numerical values indicate the percentage of sequence identity: 99.73%, 99.22%, 86.45%, 70.10%, 85.09%, 84.94%, 73.77%, and 97.02% respectively. GMQE values for each model: 0.95, 0.86, 0.62, 0.60, 0.77, 0.77, 0.86, and 0.79, respectively

To investigate sequence and structural features, representative WD40 proteins from the eight subfamilies were subjected to three-dimensional modeling. Members of each subfamily were successfully matched to a distinct GRAS protein template (Fig. 3): Subfamily I (PyWD40-28) to A0A6M2E7Y9.1.A; Subfamily II (PyWD40-65) to B9GS95.1.A; Subfamily III (PyWD40-216) to A0A6M2EW64.1.A; Subfamily IV (PyWD40-137) to A0A2K1Z0D2.1.A; Subfamily V (PyWD40-55) to A0A2K1Z638.1.A; Subfamily VI (PyWD40-99) to I1NJ39.1.A; Subfamily VII (PyWD40-85) to A0A7J7BYT5.1.A; and Subfamily VIII (PyWD40-64) to A0A4U5Q2H2.1.A..

Integrated sequence and structural analysis revealed that all modeled proteins were rich in β-sheet structures (Figure S1). The specific secondary structure composition for each model was as follows: Subfamily I: 31 β-sheets, 1 α-helix, 3 η-helices; Subfamily II: 30 β-sheets, 3 α-helices, 1 η-helix; Subfamily III: 37 β-sheets, 3 α-helices, 4 η-helices; Subfamily IV: 18 β-sheets, 1 η-helix; Subfamily V: 32 β-sheets, 1 α-helix, 3 η-helices; Subfamily VI: 62 β-sheets, 13 α-helices, 7 η-helices; Subfamily VII: 28 β-sheets, 7 α-helices; Subfamily VIII: 32 β-sheets.

### Structure analysis of the gene and protein sequences of *P. yunnanensis* WD40

**Fig. 4:**
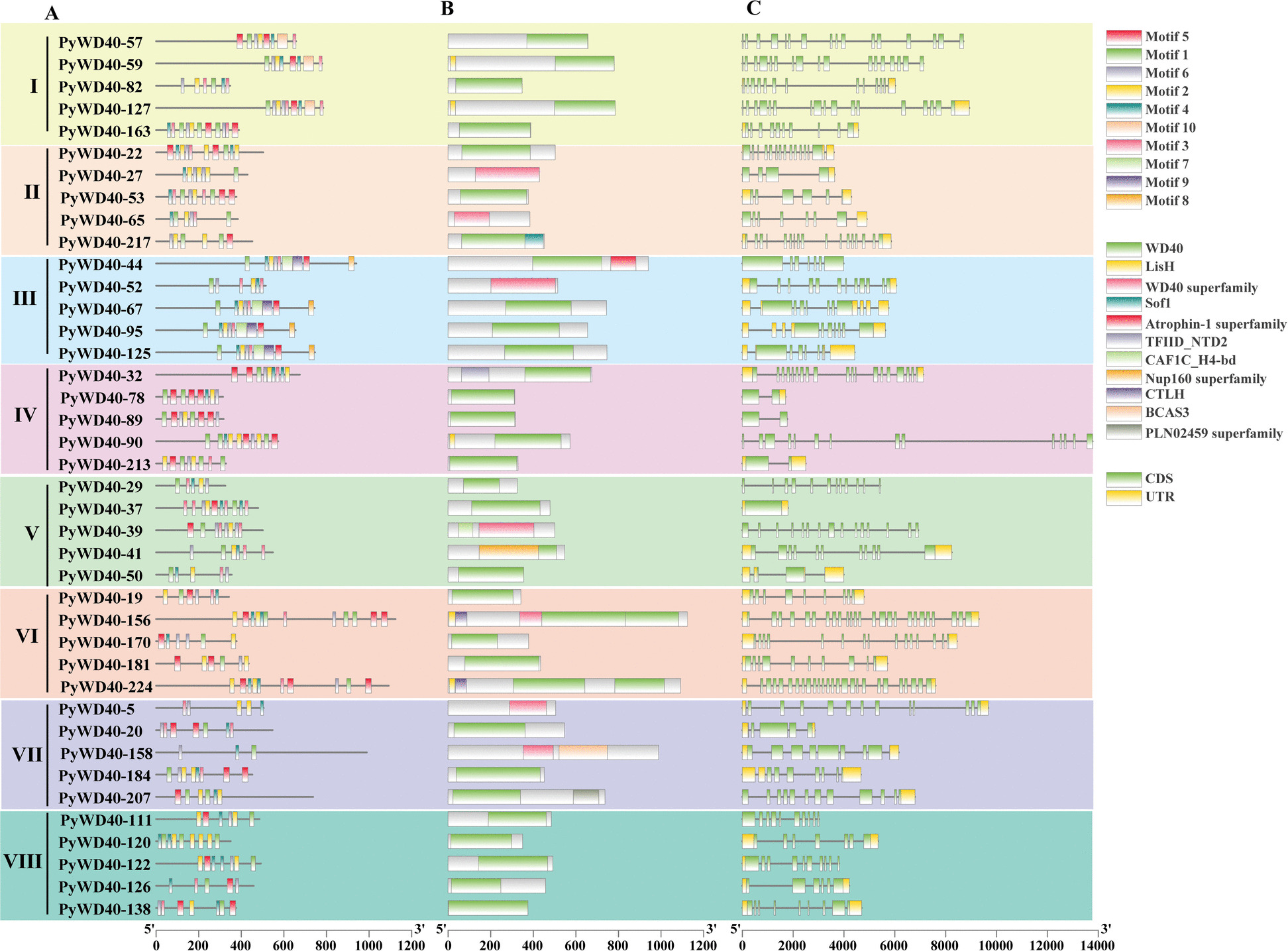
Sequence and structural features of representative WD40 proteins from *P. yunnanensis*. The figure displays an integrated analysis of (**A**) conserved motifs, (**B**) protein domains and (**C**) gene structure for representative members of the eight WD40 subfamilies (I-VIII) in *P. yunnanensis*. In panel A, different colored boxes represent distinct conserved motifs identified by MEME suite analysis. In panel B, different colored boxes indicate various conserved protein domains, as listed on the right. In panel C, green boxes represent exons, black lines represent introns, and yellow boxes represent untranslated regions (UTRs). The comprehensive figure for all 258 WD40 members is provided in Supplementary Figure S2

To further characterize the WD40 family, we analyzed their gene structures, conserved protein motifs, and domains (Fig. 4, Figure S2, Table S6). Beyond the core WD40 domain, the proteins harbored diverse auxiliary domains, the proteins harbored diverse auxiliary domains, which were classified into 27 distinct classes (Table 2). The majority of proteins (143) contained only the core WD40 domain (class 1). Numerous other proteins contained domains associated with specific functions, including metabolism (BOP1NT), vesicular transport and protein sorting (Coatomer_WDAD, COPI_C, Beach, RING_Ubox, Vps8, Clathrin), transcriptional regulation (Bromodomain, PHA03151), enzyme activity or recruitment (PH-like, Med15, BCAS3, F-box, RING_Ubox), signal transduction (CLH, PKc_like), and miRNA splicing (Prp19).

**Table 2:** Statistical table of domain types for *P. yunnanensis* WD40

Classes	Domain composition	Gene number
Class 1	OnlyWD40	143
Class 2	WD40 + CTLH or CTLH + LisH or CTLH + LisH_TPL or CTLH + LisH + Atrophin-1	16
Class 3	PLN00181 or PLN00181 + RING_Ubox	10
Class 4	WD40 + CAF1C_H4-bd	9
Class 5	WD40 + LisH or LisH + Med15	7
Class 6	WD40 + RING_Ubox or RING_Ubox + CLH or RING_Ubox + PKc_like or RING_Ubox + Vps8 + Clathrin or RING_Ubox + Prp19 or RING_Ubox + Prp19 + COG4913	7
Class 7	WD40 + Utp12 or Utp12 + ANAPC4_WD40 or Utp13 or Utp13 + PLN00181 or UTP15_C	7
Class 8	WD40 + Coatomer_WDAD or Coatomer_WDAD + COPI_C	4
Class 9	NBCH_WD40 + Beach + DUF4704 + PH-like or NBCH_WD40 + Beach + DUF4704 + PH-like + Neurobeachin + DUF4800 + Laminin_G_3	3
Class 10	WD40 + Beach + PKc_like or Beach + PH-like + Laminin_G_3 + DUF4704	3
Class 11	WD40 + Bromodomain or Bromodomain + PHA03151	3
Class 12	WD40 + Katanin_con80 or Katanin_con80 + Herpes_BLLF1	3
Class 13	WD40 + Med15	3
Class 14	WD40 + BCAS3	2
Class 15	WD40 + BING4CT	2
Class 16	WD40 + BOP1NT or BOP1NT + DMP1	2
Class 17	WD40 + DENN + uDENN + dDENN	2
Class 18	WD40 + F-box_SF or F-box-like	2
Class 19	WD40 + Hira	2
Class 20	WD40 + NLE	2
Class 21	WD40 + Nup160	2
Class 22	WD40 + PUL + PFU	2
Class 23	WD40 + Sof1	2
Class 24	WD40 + TAF5_NTD2	2
Class 25	WD40 + Ubl1_cv_Nsp3_N-like	2
Class 26	WD40 + zf-CCCH_3 or zf_CCCH_4	2
Class 27	Other	14

All identified 258 proteins contain characteristic WD repeats. Domain architecture analysis specified that members of subfamilies I, II, III, IV, V, VI, VII, and VIII were annotated with the WD40 superfamily domain (PF00400), while the remaining members (largely in subfamily VI) were annotated with the PLN00181 domain, a related WD40-like superfamily member in Pfam. Notably, the LisH domain (Lissencephaly type-1-like homology) was identified in five members of subfamily I, two of subfamily IV, ten of subfamily VI and one of subfamily V. The Med15 domain was detected in PyWD40-36 (subfamily I); the CTLH (C-terminal to LisH) domain was detected in five of subfamily II; ten of subfamily VI and one of subfamily V. The RING-Ubox domain was present in two members of subfamily II, one of subfamily V, five of subfamily VI, and one of subfamily VIII.

Motif analysis indicated that no single motifs was common to all WD40 members. However, most of the identified motifs (1, 2, 4, 5, 7, 8, 10) contained the characteristic Trp (W) and Asp (D) residues (Table S6). Motif 1, 2, 3, 4, 5, 7, 9, and 10 were the most prevalent across the subfamilies, with distinct distribution patterns (e.g., Motif 1 was prevalent in subfamily I, II, III, IV, VI; Motif 2 in I, III, IV, V, VI, and VIII; Motif 3 in II, III, V, VIII; Motif 4 in I, II, III, IV, VIII; Motif 5 in I, VI, VIII; Motif 7 in most members of subfamily I, II, III, IV, V, VII, VIII; Motif 9 in most members of subfamily I, II, III, IV, VIII; Motif 10 in most members of subfamily II, III, VII).

The exon–intron structures of *P. yunnanensis WD40* genes were highly diverse, with exon numbers ranging from 1 to 40 (Table S6). This structural heterogeneity was evident within subfamilies. For instance, Subfamily V and VI contained the most complex genes, with maximum exon counts of 25 and 32, respectively. In contrast, genes in Subfamily III predominantly had simpler architectures, with 7 to 12 exons. This variation suggests evolutionary divergence in gene structure among the WD40 subfamilies.

### Abundant *cis*-acting elements implied the functions and regulatory mechanisms of WD40 in *P. yunnanensis*

**Fig. 5:**
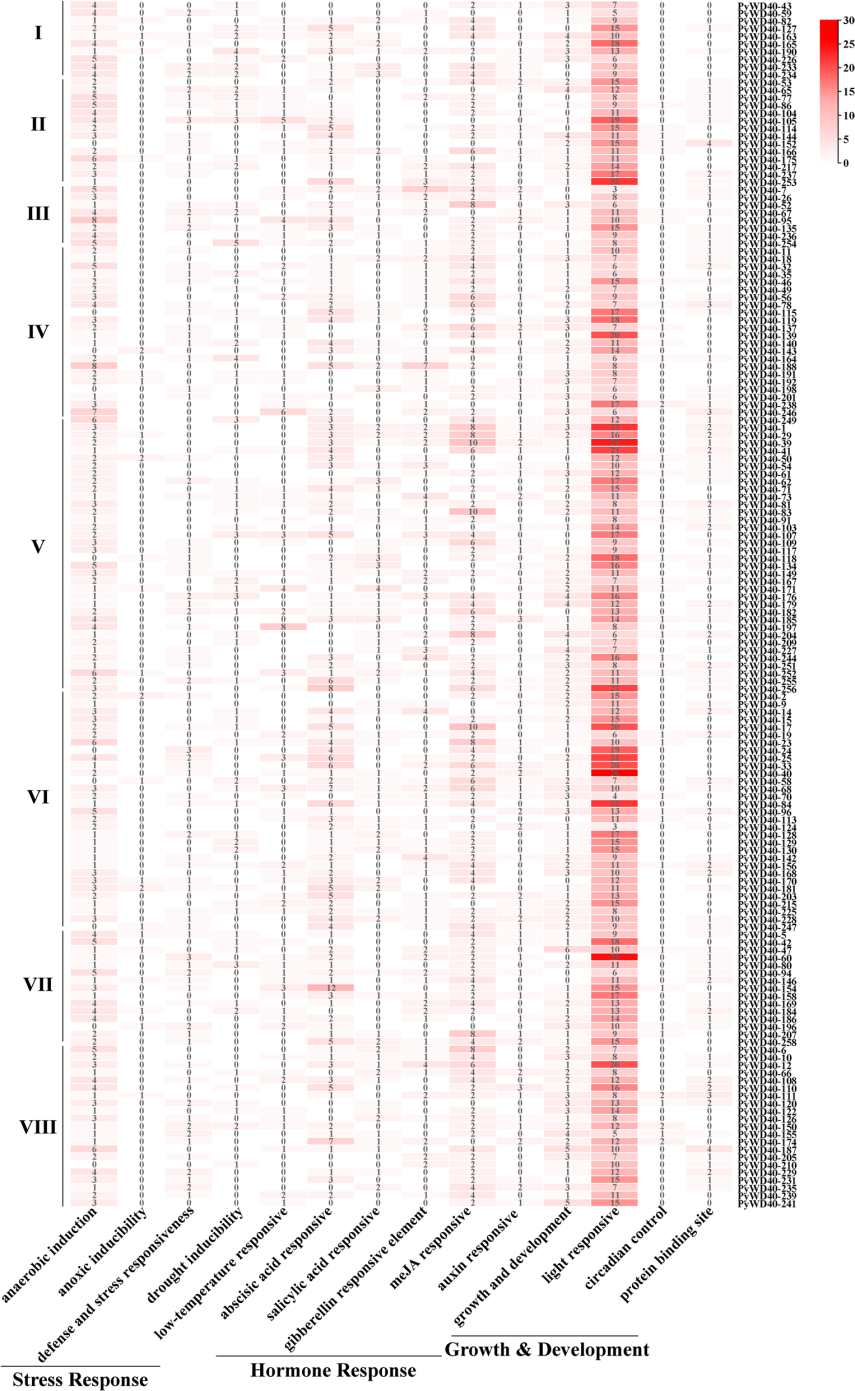
Classification and abundance of cis-acting elements in the promoters of *P. yunnanensis* *WD40* genes. Heatmap depicting the number of cis-acting elements in the 2 kb promoter regions of representative *WD40* genes from eight subfamilies (I-VIII). The elements are grouped into three major functional categories: Stress Response, Hormone Response, and Growth & Development. The color scale on the right represents the normalized count or abundance of each element type, with colors ranging from white (low abundance) to red (high abundance)

To investigate the potential roles of *P. yunnanensis WD40* genes in stress responses and their transcriptional regulation, we analyzed *cis*-acting elements within the 2-kb promoter regions upstream of each gene (Fig. 5, Table S7). A diverse array of elements was identified and categorized into three functional classes: stress-responsive (e.g., anaerobic induction, drought, low-temperature, defense), hormone-responsive (ABA, SA, GA, MeJA, auxin), and growth/development-related (light response, circadian control, protein-binding sites). Although prevalent, the abundance and combination of these elements varied markedly across subfamilies, providing a basis for functional distinction. Subfamilies II and III displayed minimalistic profiles, predominantly containing only light-responsive elements, or a combination of light-responsive and anaerobic induction elements, respectively. In contrast, Subfamilies IV-VIII possessed complex profiles, with most members harboring elements responsive to ABA, MeJA, anaerobic induction, and growth and development. Gibberellin (GA)-responsive elements were a distinctive feature exclusive to most members of Subfamilies IV and V. Subfamily I presented an intermediate and heterogeneous profile; most members contained a core set of light-responsive, anaerobic induction, and ABA-responsive elements, alongside secondary stress/hormone-related elements, while a few exceptions (*PyWD40-43*, *PyWD40-59*, *PyWD40-226*) lacked this core set, indicating intra-subfamily functional divergence.

Specific, less common *cis*-elements were also identified in subsets of genes, including those for anoxic-specific inducibility, maximal elicitor-mediated activation, conserved DNA module arrays (CMA3), sequence conserved in alpha-amylase promoter sand wound responsiveness (Table S7).

### Expression profile analysis and verification of expression status of *WD40* genes in *P. yunnanensis* under salt stress

**Fig. 6:**
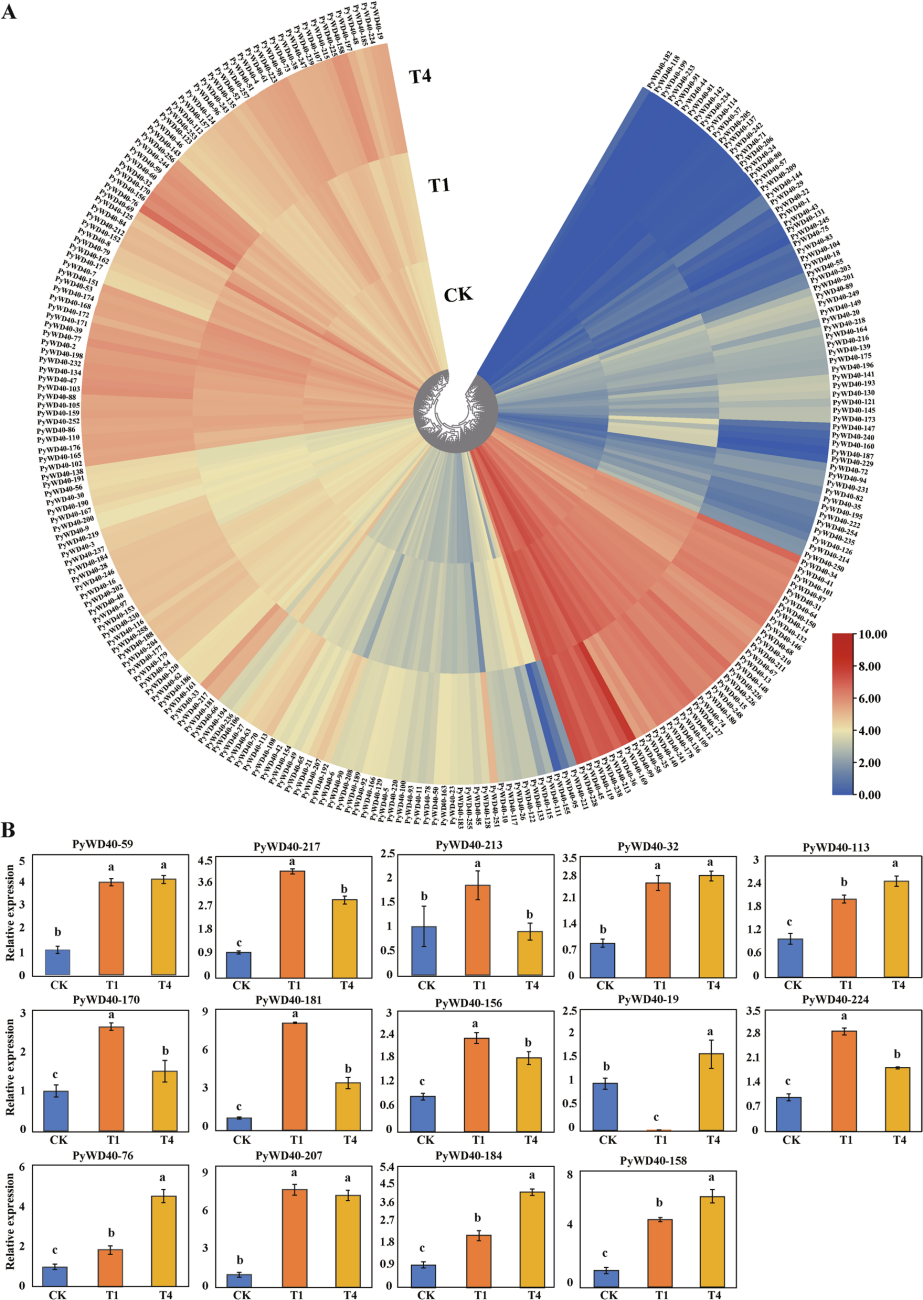
Relative expression levels of *WD40* genes from *P. yunnanensis* under salt stress. **A** Transcriptome heatmap of *P. yunnanensis WD40* genes under salt stress. CK, untreated control; T1: 25 mM NaCl treatment for 2 d; T4: 75 mM NaCl treatment for 2 d after three-round treatments. **B** Relative expression levels of *P. yunnanensis WD40* genes determined by qRT-PCR

Transcriptional profiles of *WD40* genes were analyzed under control (CK), short-term low-concentration (25 mM NaCl, T1), and long-term high-concentration (75 mM NaCl, T4) salt stress conditions using existing RNA-seq data (Table S8). Most *WD40* family members were upregulated under salt stress (Fig. 6A). While a small subset was downregulated or unchanged, the predominant upregulation indicates broad involvement in the salt stress response. Expression patterns also differed among subfamilies. Most members of subfamilies I and II, and the majority in IV-VIII, showed significant activation. Notably, the expression of many genes was basally low (FPKM < 100). Conversely, some highly expressed genes (FPKM > 100) were downregulated under salt stress, including *PyWD40-140* (IV), *PyWD40-109* (V), *PyWD40-25*, *PyWD40-58*, *PyWD40-136*, *PyWD40-178* (VI), and *PyWD40-241* (VIII).

To validate these patterns, qRT-PCR was performed on 14 representative differentially expressed *PyWD40* genes (Fig. 6B). All 14 genes (*PyWD40-19*, *PyWD40-32*, *PyWD40-59*, *PyWD40-76*, *PyWD40-113*, *PyWD40-156*, *PyWD40-158*, *PyWD40-170*, *PyWD40-181*, *PyWD40-184*, *PyWD40-207*, *PyWD40-213*, *PyWD40-217*, *PyWD40-224*) exhibited upregulated expression trends under salt stress. Most were induced by both T1 and T4 treatment, with only *PyWD40-213* and *PyWD40-19* showing specific induction under T1 or T4, respectively. These results confirmed the transcriptome data and underscore the functional diversity of *WD40* genes in the salt stress adaptation.

### Analysis of the protein interaction network of the WD40 family in *P. yunnanensis*

**Fig. 7:**
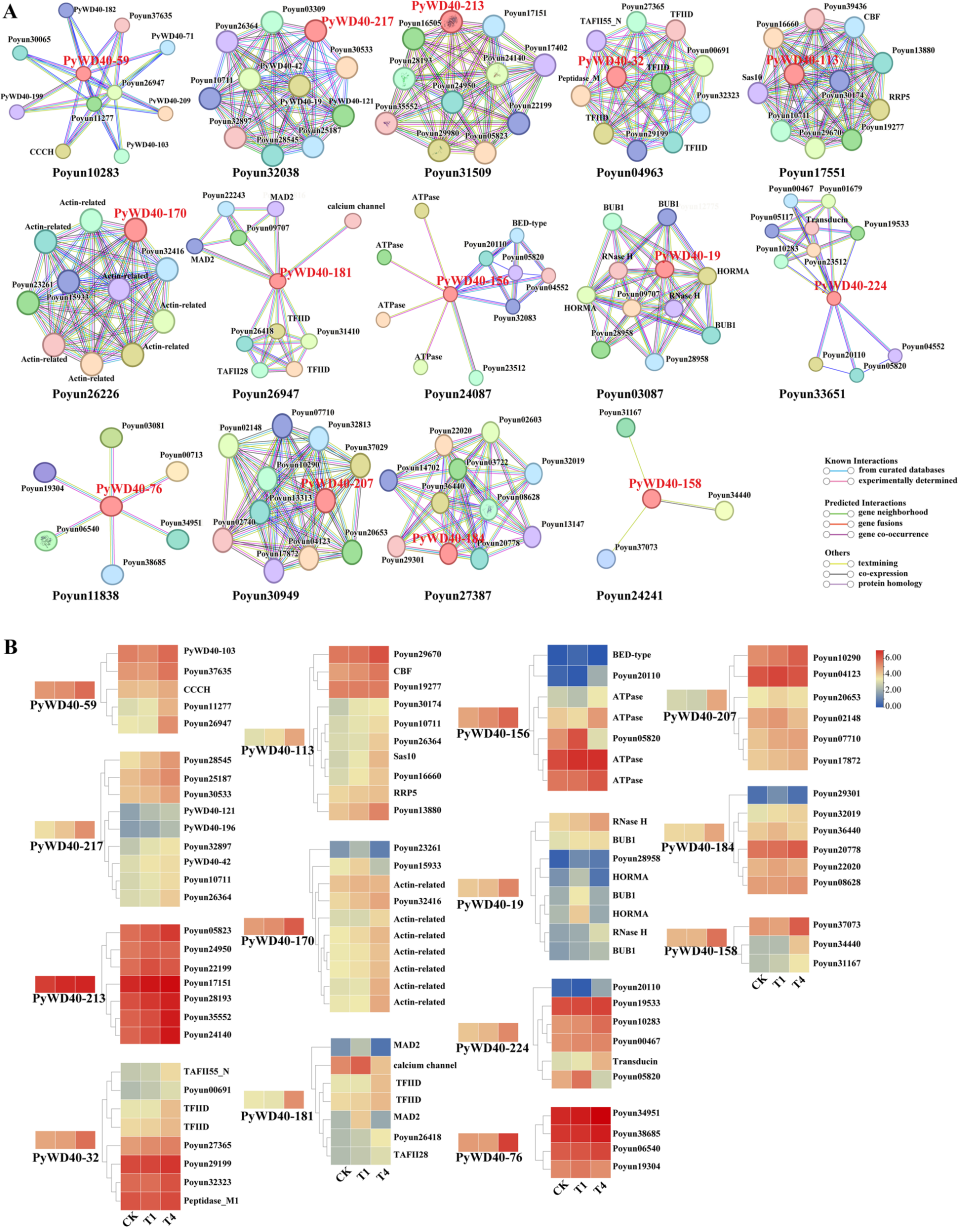
Interaction network of *P. yunnanensis* WD40 proteins and the relative expression of the interacting proteins. **A** Interaction network of *P. yunnanensis* WD40 proteins. The core-colored balls represent the representative *P. yunnanensis* WD40 proteins. The colorful balls around represent the interacting proteins of *P. yunnanensis* WD40 proteins. **B** Heatmap of the relative expression levels of the interacting proteins of *P. yunnanensis* WD40. The relative expression values were obtained from transcription data

To elucidate the regulatory network of WD40 proteins, a protein–protein interaction network was predicted for representative PyWD40s using the STRING database (Fig. 7A, Table S9). Beyond interactions with other WD40-domain proteins, PyWD40s were predicted to partner with proteins involved in diverse processes: signal transduction (e.g., transducin beta-like protein, calcium channel protein); nucleic acid metabolism/repair (RNase H, spliceosome, rRNA processing factors); energy synthesis and metabolism (ribosomal protein, rRNA biogenesis protein RRP5, nucleolar proteins, actin-related proteins, ATPase, MAD2); transcriptional regulation (transcription initiation factor TFIID subunits); and proteins with special domains (e.g., zinc finger, sas10, CBF, BED-type, RNA-binding, BUB1 N-terminal, HORMA domains). Correlation analysis revealed that the expression trends of many interacting proteins under salt stress were coordinated with those of their *PyWD40* partners (Fig. 7B, Table S10). For instance, *PyWD40-19* and its interactors (RNase H, BUB1 N-terminal, HORMA domain proteins), *PyWD40-32* and its partners (Peptidase_M1, TAFII55_N, TAFII28, TFIIDl), and *PyWD40-156* with an interacting ATPase, all showed concurrent upregulation under stress. These co-expression patterns suggested that PyWD40s may function in concert with specific partners to facilitate the salt stress response.

## Discussion

### Genomic diversity and evolutionary conservation of WD40 proteins in *Populus*

The WD domain, characterized by Trp-Asp (WD) dipeptide repeats, is the hallmark feature of this protein family [1]. Consistent with previous studies across various plant species [17, 21–28], our work confirms that WD40 protein copy number varies substantially and shows no strict correlation with genome size or complexity. This trend is evident within the genus *Populus*, where we identified between 250 and 427 WD40 proteins across six species (Table S1). In *P. yunnanensis*, the 258 identified PyWD40 proteins exhibit significant diversity in key physicochemical properties, including molecular weight, isoelectric point (pI), stability, and predicted subcellular localization (Table 1), suggesting broad functional diversification within this family [18, 21, 33].

Phylogenetic analysis classified the PyWD40 proteins into eight distinct subfamilies (Fig. 1, Table S2). The number of subfamilies differs from reports in species like tomato, walnut, and wheat [21, 26, 28], underscoring lineage-specific evolutionary paths. The evolutionary trajectory of these genes is characterized by purifying selection (Ka/Ks < 1; Table S5), and expansion via both segmental and tandem duplication events (Fig. 2, Tables S4, S5), a common mechanism for gene family diversification [34, 35]. Furthermore, strong collinearity and sequence conservation with *A. thaliana* WD40s highlight the deep conservation of this family (Fig. 2B, Table S3) [36].

### Structural and regulatory divergence underpins subfamily functional specialization

Beyond the conserved β-propeller structure that mediates protein–protein interactions [37], our analysis uncovers critical variations likely responsible for subfamily-specific functions. Although all subfamilies possess the core WD40 domain, we identified 27 distinct auxiliary domains (Table 2, Fig. 4, Figure S2). Importantly, the distribution of these domains is non-random and exhibits strong subfamily associations, providing primary evidence for functional divergence. For example, the LisH and CTLH domains are predominantly co-localized in Subfamily VI members (e.g., PyWD40-14, PyWD40-33, PyWD40-34), whereas the RING-Ubox domain is sparsely distributed across Subfamilies II, V, VI, and VIII. Given the known roles of these domains in protein dimerization, ubiquitination, and transcriptional regulation [38–40], their distinct distribution patterns imply specialized molecular functions for the respective subfamilies.

This structural divergence is paralleled by distinct regulatory landscapes. *Cis*-acting element analysis revealed minimalistic promoter profiles in subfamilies II and III, containing primarily light-responsive or combined light/anaerobic induction elements. In stark contrast, subfamilies IV through VIII possess complex promoters enriched with elements responsive to ABA, MeJA, and growth regulators; GA-responsive elements area distinctive hallmark of subfamilies IV and V. Subfamily I displays an intermediate and heterogeneous profile, where most members share a core set of stress-responsive elements but notable exceptions (e.g., *PyWD40-43*, *PyWD40-59*, *PyWD40-226*) exist, hinting at sub-functionalization (Fig. 5, Table S7). This clear partitioning of promoter architectures strongly suggests that the eight subfamilies are governed by different transcriptional programs and participate in distinct biological processes.

The phylogenetic association with functionally characterized *Arabidopsis* WD40 proteins provides compelling support for these functional predictions. For example, several PyWD40s within the clade containing Subfamily V cluster closely with *Arabidopsis* MSI1. Since AtMSI1 is a core component of the chromatin-remodeling FIS/PRC2 complex involved in gene silencing and development, this association suggests that Subfamily V members may have conserved roles in epigenetic regulation and ribosome biogenesis [41]. Similarly, the placement of stress-related genes like *Arabidopsis* XIW1 (involved in drought stress) within clades containing Subfamilies VIII members corroborates the inference from *cis*-element analysis that these subfamilies are specialized for abiotic stress response [14]. Thus, the phylogenetic tree serves not only as a classification tool but also as a functional roadmap, leveraging knowledge from model species to generate testable hypotheses about PyWD40s functions.

### Integrated expression and interaction networks support subfamily roles in salt stress adaptation

The functional relevance of this subfamily classification is corroborated by salt stress response data. Expression profiling showed that members of the "complex" subfamilies (IV-VIII) were prominently upregulated under salt stress (Fig. 6), which aligns with their enrichment for ABA- and MeJA-responsive promoter elements. The protein interaction network offers mechanistic insights: the coordinated upregulation of PyWD40s like PyWD40-19, PyWD40-32, and PyWD40-156 with predicted partners—such as transcription initiation factors (TFIID), RNase H, and ATPases—suggests that different subfamilies contribute to salt tolerance via distinct mechanisms involving transcriptional regulation, nucleic acid metabolism, and energy homeostasis (Fig. 7, Tables S9, S10) [42–44]. The heterogeneous expression patterns within Subfamily I further align with its variable *cis*-element composition, validating the predicted functional divergence.

In summary, the eight-subfamily classification of PyWD40s is not merely structural but constitutes a functionally informative framework. This framework, supported by integrated evidence from domains architecture, promoter elements, and expression dynamics, leads us to hypothesize that Subfamilies II and III are specialists in basal environmental sensing, whereas Subfamilies IV-VIII are genera lists adept at integrating complex hormonal and stress signals, particularly during abiotic stress adaptation like salinity.

## Conclusion

This study identified 258 WD40 proteins in *P. yunnanensis*, which were phylogenetically classified into eight subfamilies. These subfamilies have undergone expansion through gene duplication events and exhibit high sequence conservation. The prevalence of β-sheet structures in PyWD40 proteins supports their potential as scaffolds for protein–protein interactions. Protein interaction network analysis revealed that PyWD40s partner with proteins involved in various stress response pathways, and their co-expression under salt stress provides mechanistic clues linking PyWD40 function to specific regulatory networks and promoter elements. Collectively, these findings advance our understanding of the molecular basis for salt stress tolerance in *P. yunnanensis*.

## Material and methods

### Identification and physicochemical property analysis of WD40 protein family in *P. yunnanensis*

The hidden Markov model (HMM) profile for the WD40 domain (PF00400) was obtained from the Pfam database (http://pfam.xfam.org) [45]. Homologous WD40 proteins were identified from the whole-genome sequences of six *Populus* species (*P. yunnanensis*, *P. trichocarpa*, *P. tomentosa*, *P. euphratica*, *P. alba*, and *P. deltoides*) using HMMER3.0 software with an E-value cutoff of < 0.05 [46]. Redundant sequences were removed. The presence of the characteristic WD40 domain in all candidate proteins was further verified using the batch CD-search tool on the NCBI website (https://www.ncbi.nlm.nih.gov/) [47] and the SMART online database (https://smart.embl.de/) [48].

Physicochemical properties of the identified *P. yunnanensis* WD40 proteins, including amino acid number, molecular weight, theoretical isoelectric point (pI), instability index, aliphatic index, and grand average of hydropathicity (GRAVY) were predict using TBtools [49]. Subcellular localization was predicted using WOLF PSORT website (https://wolfpsort.hgc.jp/) [50].

### Phylogenetic analysis and classification of the WD40 family in *P. yunnanensis*

Protein sequences of WD40 members from *P. yunnanensis* and *A. thaliana* [27], were aligned using MAFFT (https://mafft.cbrc.jp/alignment/software/) [51]. A maximum likelihood (ML) phylogenetic tree was constructed with FastTree software (version 2.1.11) [52] under the LG amino acid substitution model with a CAT approximation (20 rate categories).

Tree topology was optimized using a combination of normal search, Nearest Neighbor Interchange (NNI), and Subtree Pruning and Regrafting (SPR) strategies. Branch support was assessed with Shimodaira–Hasegawa (SH)-like approximate likelihood ratio tests based on 1000 replicates. The resulting tree was visualized and annotated using the ITOL website (https://itol.embl.de/) [53], a *Chlamydomonas reinhardtii* WD40 protein Cre06.g302750_4532 was used as the outgroup (https://phytozome-ext.jgi.doe.gov/report/gene/CreinhardtiiCC_4532_v6_1/Cre06.g302750_4532). *P. yunnanensis* WD40 proteins were classified into eight subfamilies based on phylogenetic clustering and the established classification of *A. thaliana* WD40s.

### Chromosomal localization and collinearity analysis of the WD40 family in *P. yunnanensis*

Chromosomal locations of *P. yunnanensis WD40* genes were mapped using TBtools based on genome annotation data (GFF file). Intraspecific collinearity among *P. yunnanensis WD40* genes was analyzed using TBtools with the whole-genome sequence and annotation as input. Interspecific collinearity between *P. yunnanensis* and *A. thaliana WD40*s genes was also investigated using TBtools. The nonsynonymous to synonymous substitution rate ratio (Ka/Ks) for duplicated *P. yunnanensis WD40* gene pairs was calculated using TBtools.

### Protein model prediction and multiple sequence alignment of WD40 proteins in *P. yunnanensis*

Representative protein sequences from each of the eight WD40 subfamilies in *P. yunnanensis* were submitted to the SWISS-MODEL online website (https://swissmodel.expasy.org/interactive/) [54] for homology-based three-dimensional structure prediction. The resulting structural models, along with multiple sequence alignment files generated by MAFFT (see Sect. 2), were used as input for ESPript 3.0 (https://espript.ibcp.fr/ESPript/ESPript/index.php) [55] to generate figures integrating sequence alignment and secondary structure visualization.

### Analysis of conserved protein sequences, domains and genes structure of the WD40 family in *P. yunnanensis*

Conserved motifs within the WD40 protein sequences were identified using the MEME website (https://meme-suite.org/meme/tools/meme) [56], with a parameter setting to discover up to 10 motifs. Protein domains were predicted using the Batch CD-search tool on the NCBI website (https://www.ncbi.nlm.nih.gov/Structure/bwrpsb/bwrpsb.cgi). Gene structure diagrams (exon–intron organization) and integrated visualizations combining motif, domain, and gene structure information were generated using TBtools based on the *P. yunnanensis* genome annotation (GFF3 file).

### Prediction of *cis*-acting elements within the WD40 family of *P. yunnanensis*

The 2000 bp genomic sequences upstream of the translation start site of each *WD40* genes were extracted using TBtools. *Cis*-acting regulatory elements within these promoter regions were predicted using the PlantCARE website (https://bioinformatics.psb.ugent.be/webtools/plantcare/html/) [57]. Results were visualized using TBtools.

### Expression profile analysis of the WD40 family in *P. yunnanensis* under salt stress and quantitative real-time PCR (qRT-PCR)

Transcript expression levels (FPKM values) for *WD40* genes were obtained from a previously published RNA-seq dataset (NCBI BioProject: PRJNA998345) generated from *P. yunnanensis* leaves under control (CK), short-term low-concentration salt stress (T1: 25 mM NaCl, 2 d), and long-term high-concentration salt stress (T4: 75 mM NaCl, 2 d) conditions [58]. A heatmap was generated based on log2- transformed FPKM values using TBtools.

To validate the RNA-seq results, total RNA was isolated from leaf samples (CK, T1, T4) using a Plant Total RNA Extraction Kit (TIANGEN, DP190813, Beijing, China). First-strand cDNA was synthesized using a commercial reverse transcription kit (TIANGEN, AT321, Beijing, China). qRT-PCR was performed on selected differentially expressed *WD40* genes using gene-specific primers (Table S11). The *P. yunnanensis* gene *Poyun22323*, identified as a stable reference from the transcriptome data, was used as an internal control [59].

### Protein–protein interaction network analysis of the WD40 family in *P. yunnanensis*

Representative *P. yunnanensis* WD40 protein sequences were submitted to the STRING database (https://cn.string-db.org/) [60] to predict potential protein–protein interactions. Predictions were restricted to the *Populus* taxon, and only interactions with the highest confidence score were retained for further analysis.

## Supplementary Information

Supplementary Material 1.

Supplementary Material 2.

Supplementary Material 3.

Supplementary Material 4.

Supplementary Material 5.

Supplementary Material 6.

Supplementary Material 7.

Supplementary Material 8.

Supplementary Material 9.

Supplementary Material 10.

Supplementary Material 11.

Supplementary Material 12.

## Data Availability

The transcriptome data of *P*. *yunnanensis* used in this study are publicly available in the NCBI repository (National Center for Biotechnology Information, https://www.ncbi.nlm.nih.gov/), with the accession number PRJNA998345.

## Abbreviations

WD: Trp-Asp
Ip: Isoelectric points
GRAVY: Grand average of hydropathicity
LisH: Lissencephaly type-1-like homology
MeJA: Methyl Jasmonate
ABA: Abscisic acid
GA: Gibberellin
SA: Salicylic acid
qRT-PCR: Quantitative Reverse Transcription Polymerase Chain Reaction
